# Identification of extracellular surface-layer associated proteins in *Lactobacillus acidophilus* NCFM

**DOI:** 10.1099/mic.0.070755-0

**Published:** 2013-11

**Authors:** Brant Johnson, Kurt Selle, Sarah O’Flaherty, Yong Jun Goh, Todd Klaenhammer

**Affiliations:** 1 Department of Microbiology, North Carolina State University, Raleigh, NC, USA; 2 Department of Food, Bioprocessing and Nutrition Sciences, North Carolina State University, Raleigh, NC, USA

## Abstract

Bacterial surface (S-) layers are crystalline arrays of self-assembling, proteinaceous subunits called S-layer proteins (Slps), with molecular masses ranging from 40 to 200 kDa. The S-layer-forming bacterium *Lactobacillus acidophilus* NCFM expresses three major Slps: SlpA (46 kDa), SlpB (47 kDa) and SlpX (51 kDa). SlpA has a demonstrated role in adhesion to Caco-2 intestinal epithelial cells *in vitro*, and has been shown to modulate dendritic cell (DC) and T-cell functionalities with murine DCs. In this study, a modification of a standard lithium chloride S-layer extraction revealed 37 proteins were solubilized from the S-layer wash fraction. Of these, 30 have predicted cleavage sites for secretion, 24 are predicted to be extracellular, six are lipid-anchored, three have N-terminal hydrophobic membrane spanning regions and four are intracellular, potentially moonlighting proteins. Some of these proteins, designated S-layer associated proteins (SLAPs), may be loosely associated with or embedded within the bacterial S-layer complex. *Lba-1029*, a putative SLAP gene, was deleted from the chromosome of *L. acidophilus*. Phenotypic characterization of the deletion mutant demonstrated that the SLAP LBA1029 contributes to a pro-inflammatory TNF-α response from murine DCs. This study identified extracellular proteins and putative SLAPs of *L. acidophilus* NCFM using LC-MS/MS. SLAPs appear to impart important surface display features and immunological properties to microbes that are coated by S-layers.

## Introduction

Bacterial surface (S-) layers are crystalline arrays of self-assembling, proteinaceous subunits called S-layer proteins (Slps), with molecular masses ranging from 40 to 200 kDa (Sára & Sleytr, 2000). Present as the outermost component of the cell wall, S-layers are found in many species of eubacteria and archaea, and in varying environments. S-layer lattices are 5–25 nm thick and form oblique (p1, p2), square (p4) or hexagonal (p3, p6) symmetries, as observed by freeze-etched electron microscopy (Sleytr *et al.*, 2001). Further structural observations have revealed that S-layers are highly porous in nature, with pores occupying up to 70 % of the cell surface (Sleytr & Beveridge, 1999). Previous work comparing amino acid sequences from different S-layer-forming bacteria has shown that most S-layers are high in hydrophobic and acidic amino acids (Sára & Sleytr, 1996; Sleytr & Messner, 1983). Furthermore, the isoelectric points (pI) of many Slps are low, mostly found in the weakly acidic pH range (Sára & Sleytr, 2000). These sequence analyses have also revealed the presence of S-layer homologous (SLH) motifs on the N-terminal section of many Slps (Sára & Sleytr, 2000), which are responsible for tethering the S-layer to the secondary cell-wall polysaccharide (Brechtel & Bahl, 1999; Chauvaux *et al.*, 1999).

Lactic acid bacteria of the genus *Lactobacillus* are a diverse group of Gram-positive, anaerobic/microaerophilic, non-sporulating, low G+C content bacteria belonging to the phylum *Firmicutes* (Pot *et al.*, 1994). Biochemically, they are strictly fermentative; sugar fermentations result in either the sole production of lactic acid, or the production of lactic acid in conjunction with smaller amounts of carbon dioxide and ethanol/acetic acid (Hammes & Vogel, 1995; Pot *et al.*, 1994). There are several species of *Lactobacillus* that form S-layers, including mucosal-associated species (e.g. *Lactobacillus acidophilus*, *Lactobacillus crispatus*, *Lactobacillus amylovorus* and *Lactobacillus gallinarum*) and dairy fermentation-associated species (e.g. *Lactobacillus helveticus* and *Lactobacillus kefiranofaciens*) (Åvall-Jääskeläinen & Palva, 2005; Hynönen & Palva, 2013). Compared with S-layers of other Gram-positive bacteria, those from *Lactobacillus* are biochemically unique. Specifically, S-layers from *Lactobacillus* do not possess SLH domains (Boot & Pouwels, 1996). Furthermore, their Slps are among the smallest known (25–71 kDa) and are highly basic with calculated pI values ranging from 9.35 to 10.4 (Åvall-Jääskeläinen & Palva, 2005).

*L. acidophilus* NCFM is a widely used probiotic microbe, found in both fermented dairy products and dietary supplements (Sanders & Klaenhammer, 2001). Utilizing a completely sequenced and annotated genome (Altermann *et al.*, 2005), *L. acidophilus* NCFM has been the subject of many investigations seeking to understand the mechanisms of probiotic functionality. *L. acidophilus* NCFM forms an S-layer composed principally of SlpA, with auxiliary components SlpB and SlpX (Altermann *et al.*, 2005; Goh *et al.*, 2009). Given its proximity to the cell surface, the S-layer is one of the first bacterial components to interact with the gastrointestinal surface of the human host. SlpA of *L. acidophilus* NCFM has demonstrated an important role in adhesion to the Caco-2 intestinal cell line (Buck *et al.*, 2005) and has been shown to modulate dendritic cell (DC) and T-cell functionality (Konstantinov *et al.*, 2008). Taken together, these studies highlight the potential role of S-layers in probiotic activities.

Transmission electron microscopy images of *L. acidophilus* reveal a cell envelope that is abundant with S-layer components. In fact, ~ 5×10^5^ Slp subunits are required to generate the S-layer of rod-shaped cells, such as *L. acidophilus* (Sleytr & Messner, 1983). Slps have been extracted from Gram-positive bacterial cell surfaces via treatment with high concentrations of salts [e.g. guanidine hydrochloride or lithium chloride (LiCl)], which disrupt hydrogen bonding between the S-layer and the secondary cell-wall polysaccharide (Sára & Sleytr, 2000). Specifically, LiCl treatments at 5 and 1 M concentrations have been used to isolate the Slps from many *Lactobacillus* species (Ashida *et al.*, 2011; Beganović *et al.*, 2011; Frece *et al*., 2005; Goh *et al.*, 2009; Lortal *et al.*, 1992; Smit *et al.*, 2001; Taverniti *et al.*, 2013). Despite the dramatic presence of the S-layer and the highly expressed Slp subunits, it is notable that research on exoproteins associated with the S-layer is scarce. Although proteins associated with the S-layer have been observed in *L. acidophilus* (Smit *et al.*, 2001), there has been no work identifying these proteins using MS.

**Fig. 1.**
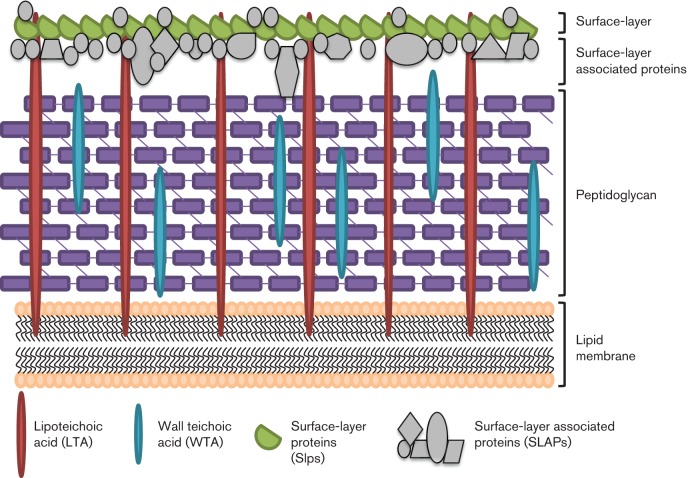
Proposed schematic for the localization of SLAPs in *L. acidophilus* NCFM. The Gram-positive bacterial cell wall is comprised of a thick peptidoglycan layer (purple), stabilized by teichoic acids and tethered to the lipid membrane by lipoteichoic acid. The S-layer, composed of self-assembling Slps (green), is the outermost layer of the cell wall. SLAPs (grey) may be associated with this outermost S-layer.

To identify these proteins in *L. acidophilus*, a LiCl S-layer extraction protocol noted above (Goh *et al.*, 2009; Lortal *et al.*, 1992) was modified to isolate proteins associated with the S-layer, while mostly excluding the major Slps. After protein identification through LC-MS/MS, 37 proteins were identified that may be associated with or embedded within the S-layer. Many of these proteins, designated S-layer associated proteins (SLAPs, Fig. 1), have unknown function and offer potential in advancing our understanding of the probiotic mechanism, cell envelope biology and immunomodulation in *L. acidophilus.* One of the predicted SLAPs, encoded by *lba-1029*, was deleted from the chromosome of *L. acidophilus* NCFM using a *upp-*based counterselective gene replacement system (Goh *et al.*, 2009). This study aimed to (i) develop a modified method for extracting SLAPs, (ii) identify the SLAPs found in *L. acidophilus* NCFM and (iii) functionally characterize the SLAP LBA1029.

## Methods

### 

#### Bacterial strains and growth conditions.

Bacterial strains, plasmids and primers used in this study are reported in Table 1. *L. acidophilus* strains were propagated in de Man Rogosa Sharpe (MRS) broth (Difco) under aerobic conditions, statically or on MRS solid medium containing 1.5 % (w/v) agar (Difco) under anaerobic conditions at 37 °C, and at 42 °C where indicated. Recombinant strains were selected with 2 µg erythromycin ml^−1^ (Sigma-Aldrich) and/or 5 µg chloramphenicol ml^−1^ (Sigma-Aldrich). *Escherichia coli* strains were grown in brain heart infusion (Difco) medium at 37 °C with shaking for aeration. *E. coli* EC101 was grown in the presence of 40 µg kanamycin ml^−1^ (Sigma-Aldrich) while NCK1911 and transformants were grown with 40 µg kanamycin ml^−1^ and 150 µg erythromycin ml^−1^. Counterselection of plasmid-free excision recombinants was performed using 5-fluorouracil-supplemented glucose semi-defined medium, as previously described (Goh *et al.*, 2009). 

**Table 1.** Strains, plasmids and primers used in this study

Strain, plasmid or primer	Genotype or characteristics	Reference
***L. acidophilus* strains**		
NCFM (NCK56)	Human intestinal isolate	(Sanders & Klaenhammer, 2001)
NCK1909	NCFM with chromosomal deletion of *upp*; background host for *upp*-based counterselective gene replacement system	(Goh et al., 2009)
NCK1910	RepA^+^, pWV01 integrated into the host chromosome. Cm^r^	(Goh et al., 2009)
NCK2258	NCK1909 with chromosomal deletion of *lba-1029*	This study
***E. coli* (EC101) strains**		
NCK1911	Host harbouring pTRK935, Kn^r^ Em^r^	(Goh et al., 2009)
NCK2257	Host harbouring pTRK1067, Kn^r^ Em^r^	This study
**Plasmids**		
pTRK669	Ori (pWV01), Cm^r^, RepA^+^ thermosensitive	(Russell & Klaenhammer, 2001)
pTRK935	pORI *upp*-based counterselective integration vector	(Goh et al., 2009)
pTRK1067	pTRK935 with flanking regions of *lba-1029* cloned into *Bam*HI and *Sac*I restriction sites	This study
Primers*		
1*Bam*HI-F	GTAATAGGATCCGCAGAAATTAAGCCCGTTGT	This study
2R	TGCAATTGTAGCCAAAATTAGTG	This study
3Soe	TAATTTTGGCTACAATTGCACACACTGCTGTTTACGATCCA	This study
4*Sac*I-R	TAAAGTAGAGCTCATCTTGCCCAATCGTGTAAA	This study
1029up	CTTAATTCACTGGCCAAATC	This study
1029dw	TCTGCTGACTTCTCTTGAGG	This study

* Restriction sites are underlined.

#### LiCl extraction of SLAPs.

The extraction protocol for SLAPs was modified from a standard LiCl S-layer extraction protocol for *L. acidophilus* (Goh *et al.*, 2009; Lortal *et al.*, 1992). Bacterial cells were grown in 200 ml MRS to stationary phase (16 h), centrifuged at 2236 ***g*** for 10 min (4 °C), and washed twice with 25 ml cold PBS (Gibco), pH 7.4. Cells were agitated for 15 min at 4 °C following the addition of 5 M LiCl (Fisher Scientific). Supernatants, containing Slps and SLAPs, were harvested via centrifugation at 8994 ***g*** for 10 min (4 °C) and transferred to a 6000–8000 kDa Spectra/Por molecular porous membrane (Spectrum Laboratories) and dialysed against cold distilled water for 24 h, changing the water every 2 h for the first 8 h. The dialysed precipitate was harvested via centrifugation at 20 000 ***g*** for 30 min and agitated for a second time for 15 min with 1 M LiCl at 4 °C to disassociate the SLAPs from the Slps, which are insoluble in 1 M LiCl. Next, the suspension was centrifuged at 20 000 ***g*** for 10 min and the supernatants, containing the SLAPs, were again transferred to the 6000–8000 kDa Spectra/Por molecular porous membrane and dialysed against cold distilled water for 24 h. Finally, the precipitate was harvested via centrifugation at 20 000 ***g*** for 30 min and pellets were resuspended in 10 % (w/v) SDS (Fisher). Proteins were quantified via bicinchoninic acid assay kit (Thermo Scientific) and visualized via SDS-PAGE using precast 4–20 % Precise Tris-HEPES protein gels (Thermo Scientific). The gels were stained using AcquaStain (Bulldog Bio) according to the instructions of the manufacturer.

#### Protein identification.

The SLAPs were electrophoresed for approximately 7 min in the resolving gel of the SDS-PAGE and excised using a sterile blade. The protein gel was submitted to the Genome Center Proteomics Core at the University of California, Davis, for MS-based protein identification. Briefly, proteins were reduced and alkylated according to the procedures of Shevchenko *et al.* (1996), and digested with sequencing-grade tryspin according to the manufacturer’s instructions (Promega). Peptides were dried down in a vacuum concentrator after digestion and then resolubilized in 2 % acetonitrile/0.1 % trifluoroacetic acid. Digested peptides were analysed by LC-MS/MS on a Thermo Scientific Q Exactive Orbitrap mass spectrometer in conjunction with a Paradigm MG4 HPLC machine and CTC Pal auto sampler (Michrom Bio Resources). The digested peptides were loaded onto a Michrom C18 trap and desalted before they were separated using a Michrom 200 µm×150 mm Magic C_18_AQ reversed-phase column. A flow rate of 2 µl min^−1^ was used. Peptides were eluted using a 120 min gradient with 2 % acetonitrile to 35 % acetonitrile over 94 min, 35 % acetonitrile to 80 % acetonitrile for 10 min, 80 % acetonitrile for 2 min, and then a decrease from 80 to 5 % acetonitrile in 1 min. An MS survey scan was obtained for the *m*/*z* range 300–1600. MS/MS spectra were acquired using a top 15 method, where the top 15 ions in the MS spectra were subjected to high energy collisional dissociation. An isolation mass window of 2.0 *m*/*z* was used for the precursor ion selection, and a normalized collision energy of 27 % was used for fragmentation. A 5 s duration was used for the dynamic exclusion.

#### Protein database searches.

Tandem mass spectra were extracted and charge state deconvoluted using MM File Conversion version 3. All MS/MS samples were analysed using X! Tandem (The GPM, www.thegpm.org/; version TORNADO). X! Tandem was set up to search the Uniprot *L. acidophilus* database (July 2012, 1859 entries), the cRAP database of common laboratory contaminants (www.thegpm.org/crap; 114 entries) plus an equal number of reverse protein sequences (assuming the digestion enzyme trypsin). X! Tandem was searched with a fragment ion mass tolerance of 20 p.p.m. and a parent ion tolerance of 20 p.p.m. The iodoacetamide derivative of cysteine was specified in X! Tandem as a fixed modification. Deamidation of asparagine and glutamine, oxidation of methionine and tryptophan, sulfone of methionine, tryptophan oxidation to formylkynurenin of tryptophan and acetylation of the N terminus were specified in X! Tandem as variable modifications. LocateP and the LAB-Secretome database were used to predict the secretion pathways of identified proteins (Zhou *et al.*, 2008, 2010). SignalP 4.1 was used to predict the signal peptidase cleavage site of each protein (Petersen *et al.*, 2011). Grand average of hydropathicity (GRAVY) scores were predicted using the GRAVY calculator (http://www.gravy-calculator.de).

#### Criteria for protein identification.

Scaffold (version Scaffold_3.6.1, Proteome Software) was used to validate MS/MS-based peptide and protein identifications. Peptide identifications were accepted if they exceeded specific database search engine thresholds. X! Tandem identifications required scores of greater than 1.2 with a mass accuracy of 5 p.p.m. Protein identifications were accepted if they contained at least two identified peptides. Using the parameters above, the false discovery rate was calculated to be 1.1 % on the protein level and 0 % on the peptide level (Tabb *et al.*, 2008). Proteins that contained similar peptides and could not be differentiated based on MS/MS analysis alone were grouped to satisfy the principles of parsimony. For this study, only proteins with unique spectral counts of ≥10 were considered significant.

#### Molecular techniques.

Genomic DNA from *L. acidophilus* strains was isolated using a Zymo Research Fungal/Bacterial DNA MiniPrep kit (Zymo Research). Plasmid DNA from *E. coli* was isolated using a QIAprep Spin Miniprep kit (Qiagen). Restriction enzyme digestions and ligations were performed using Roche restriction enzymes (Roche Diagnostics) and T4 DNA ligase (New England Biolabs), respectively. PCR primers were designed based on the genomic sequence data and synthesized by Integrated DNA Technologies. PCRs were carried out in Bio-Rad MyCycler thermocyclers (Bio-Rad Laboratories) using Choice-*Taq* Blue DNA polymerase (Denville Scientific) for screening of recombinants and *PfuUltra* II fusion HS DNA polymerase (Agilent Technologies) for cloning purposes. PCR amplicons were analysed on 0.8 % agarose gels and purified using QIAquick Gel Extraction kits (Qiagen). DNA sequencing was performed by Davis Sequencing.

*E. coli* EC101 cells were made competent using a rubidium chloride competent cell protocol (Hanahan, 1983). *L. acidophilus* cells were prepared for electrotransformation using a modified penicillin treatment protocol (Goh *et al.*, 2009; Walker *et al.*, 1996; Wei *et al.*, 1995).

#### Sequence analysis.

Identified protein sequences were compared against the non-redundant protein database using blastp (http://blast.ncbi.nlm.nih.gov/Blast.cgi). Further protein alignments were performed by comparing the deduced protein sequence with those from all of the protein databases of the following organisms: *L. helveticus*, *L. crispatus*, *L. amylovorus*, *Lactobacillus gasseri*, *Lactobacillus johnsonii* and *Lactobacillus delbrueckii* subsp. *bulgaricus*. All blast analyses were performed with adjusted algorithms to display only alignments with an E-value of ≤1×10^−6^. Rho-independent transcriptional terminators were predicted by TransTermHP (Kingsford *et al.*, 2007).

#### Construction of an *L. acidophilus* Δ*lba-1029* mutant.

The *upp-*based counterselection gene replacement method, described previously (Goh *et al.*, 2009), was used as a strategy for creating an internal deletion of 1155 bp in *lba-1029* of NCK1909, a *upp*-deficient background strain of *L. acidophilus* NCFM. Using splicing by overlap extension PCR (Horton *et al.*, 1989), the 2 kb flanking regions of the deletion target were spliced with *Bam*HI restricted site added on the upstream end and *Sac*I on the downstream end. This construct was digested with *Bam*HI and *Sac*I, then ligated into the polylinker of the similarly digested integration plasmid pTRK935 and transformed into competent *E. coli* EC101. The resulting recombinant plasmid, pTRK1067, was transformed into *L. acidophilus* NCK1909 harbouring the helper plasmid pTRK669 (NCK1910). Single crossover integrants were screened as described previously (Goh *et al.*, 2009). Colonies with the Δ*lba-1029* genotype were screened among the double recombinants recovered on 5-fluorouracil glucose semi-defined medium agar plates. Deletion of *lba-1029* was confirmed by PCR and sequencing.

#### Caco-2 intestinal epithelial cells adherence assay.

For cell adherence assays, the Caco-2 intestinal epithelial cell line (ATCC HTB-37; American Type Culture Collection) was used. Cell culture media and reagents were purchased from Gibco and the assay protocol was performed as described previously (Goh *et al.*, 2009). Caco-2 cultures were grown at 37 °C in a 95 % air/5 % CO_2_ atmosphere. A minimal essential medium (MEM) supplemented with 1 mM sodium pyruvate, 20 % (v/v) heat-inactivated FBS, 0.1 mM non-essential amino acids, penicillin G (100 mg ml^−1^), streptomycin sulfate (100 mg ml^−1^) and amphotericin (0.25 mg ml^−1^) was used. Monolayers for the adhesion assay were prepared in 12-well tissue culture plates by seeding approximately 6.5×10^4^ cells per well in 2 ml cell culture medium. The culture medium was replaced every 2 days, while the monolayers were used for the assay 2 weeks post-confluence. On the day of the assay, monolayers were washed twice with 1 ml PBS before adding 1 ml MEM without antibiotics and incubating at 37 °C in a 5 % CO_2_ incubator prior to adding bacterial cells. Overnight bacterial cultures (10 ml) were pelleted via centrifugation (3166 ***g***, 10 min) at room temperature, washed and resuspended in PBS to a final concentration of ~1×10^8^ c.f.u. ml^−1^. Next, 1 ml of the bacterial suspension was added to each well of the cell monolayer in triplicate and incubated at 37 °C in a 5 % CO_2_ atmosphere for 1 h. Following incubation, monolayers were washed five times with 1 ml PBS and treated with 1 ml 0.05 % (v/v) Triton X-100. After 10 min at 37 °C, cell monolayers were disrupted via pipetting and transferred to microcentrifuge tubes. Finally, microbial cells from the monolayer were diluted and enumerated after plating onto MRS agar.

#### Simulated gastric and small intestinal juice assays.

Simulated gastric juice and small intestinal juice were prepared as previously described (Goh & Klaenhammer, 2010). Overnight cultures were centrifuged, washed twice in PBS and resuspended in sterile distilled water. The cell suspension (1.2 ml) was mixed with 6 ml of freshly prepared simulated gastric juice [0.5 % (w/v) NaCl solution with 3 g pepsin l^−1^ (Fisher Scientific), pH adjusted to 2.0 with HCl] or simulated small intestinal juice [0.5 % (w/v) NaCl solution containing 1 g pancreatin l^−1^ (Sigma) and 3 g Oxgall l^−1^ (Difco), pH adjusted to 8.0 with NaOH] and incubated at 37 °C. Viable cell counts were determined by plating onto MRS agar after 30 min, 1 h and 1.5 h in simulated gastric juice, and hourly for 5 h in simulated small intestinal juice.

#### Bile tolerance assays.

Cells were tested for bile tolerance in two separate assays. First, cells were inoculated into a 96-well plate containing 200 µl per well of MRS, MRS+0.3 % (w/v) Oxgall or MRS+0.5 % Oxgall in triplicates. Growth curves were monitored over 24 h by measuring the absorbance (OD_600_) using a FLUOStar Optima microtitre plate reader (BMG Labtech). Secondly, cells were measured in planktonic growth in 10 ml of each of the three media above. At each time point, OD_600_ was measured and cells were plated for c.f.u. enumeration on MRS agar.

#### Bacterial-DC co-incubation and cytokine measurement.

An *in vitro* DC co-incubation assay was performed based on a modification of previous protocols (Mohamadzadeh *et al.*, 2011; Stoeker *et al.*, 2011). Bone marrow-derived BALB/c murine immature DCs (iDCs) were acquired (Astarte-Biologics) and preserved in liquid nitrogen. On the day of the assay, iDCs were thawed in a 37 °C water bath and transferred to a 50 ml conical tube containing 100 µg of DNase I at a concentration of 1 mg ml^−1^ (Stem Cell Technologies) to prevent clumping. RPMI 1640 medium with 10 % FBS was added to the DCs, which were subsequently centrifuged in a swing arm rotor (200 ***g***) at room temperature for 10 min. An aliquot of cells was removed for enumeration of live cells using Trypan Blue (Sigma) and the Invitrogen Countess, according to the manufacturer’s instructions. Viable cells were then diluted to a final concentration of 1×10^6^ ml^−1^ in the RPMI 1640+10 % FBS + 100 µg streptomycin ml^−1^ and aliquoted (100 µl per well) into round bottom polypropylene 96-well plates and held in 5 % CO_2_ at 37 °C. Bacterial strains grown to stationary phase (16 h) were harvested by centrifugation, washed, resuspended in PBS and then standardized to ~1×10^8^ c.f.u. ml^−1^. A portion of these aliquots was set aside for dilution and enumerative plating on MRS agar. The standardized cell suspension was centrifuged and resuspended in RPMI 1640+10 % FBS + 100 ug streptomycin ml-1 and 1×10^6^ cells were combined with 1×10^5^ viable iDCs in each well, resulting in a final bacterial to DC ratio of 10 : 1. The bacterial cells and iDCs were co-incubated for 24 h in 5 % CO_2_ at 37 °C, after which the suspension was centrifuged and the supernatant was harvested and stored at −80 °C for cytokine analysis.

Cytokine measurements for TNF-α, IL-6, IL-10 and IL-12 were quantified using Single-Analyte ELISArray kits (Qiagen), according to the manufacturer’s instructions. Following cytokine quantification, the cytokine expression data were compared between the parent and mutant strains using univariate analysis of variance.

## Results

### Identification of extracellular and putative SLAPs from modified LiCl extraction

**Fig. 2.**
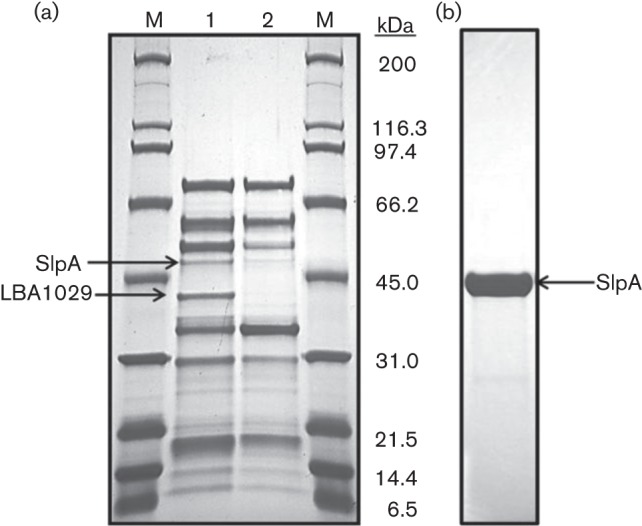
(a) Putative SLAPs of *L. acidophilus* NCFM (NCK56) and NCK2258. Proteins were extracted using a series of washes in LiCl followed by dialyses in molecular porous membranes at 4 °C. The relative molecular masses (M) are labelled. Lane 1, SLAPs from *L. acidophilus* NCFM; lane 2, SLAPs from NCK2258, demonstrating the absence of the 43 kDa LBA1029. (b) Pure SlpA from *L. acidophilus* NCFM. Note the absence of other potential SLAPs/extracellular proteins using the standard protocol.

Previous work characterizing the S-layer of lactobacilli used 5 M LiCl salt to extract Slps efficiently with less lethality than other denaturing salts, such as guanidine hydrochloride (Ashida *et al.*, 2011; Beganović *et al.*, 2011; Goh *et al.*, 2009; Lortal *et al.*, 1992; Smit *et al.*, 2001; Taverniti *et al.*, 2013). To investigate the potential presence of SLAPs, a LiCl S-layer extraction protocol (Goh *et al.*, 2009; Lortal *et al.*, 1992) was modified. Cells were treated with 5 M LiCl, solubilizing all non-covalently bound Slp and proteins associated with the S-layer, and the proteins were extracted via dialysis. This fraction was then treated with 1 M LiCl to separate the Slp from the proteins associated with the S-layer. These supernatants, containing only the proteins associated with the S-layer, were centrifuged, dialysed and electrophoresed on SDS-PAGE (Fig. 2, lane 1). This subset of proteins may be associated with or embedded within the S-layer of *L. acidophilus* NCFM.

Proteins from the modified LiCl extraction protocol were identified using LC-MS/MS following trypsin digestion. After database searching, the identified proteins were presented using Scaffold proteome software. Proteomic data were processed by removing human contaminants (e.g. keratin), falsely identified mammalian proteins and any protein with a unique spectral count ≤10, leaving 37 proteins of interest with molecular masses ranging from 10 to 78 kDa (Table 2). The newly identified SLAPs were ordered from the highest to lowest unique spectral counts. Interestingly, GRAVY values predicted that all proteins of interest were hydrophilic in nature. Secretion of proteins in Gram-positive bacteria is mediated through the Sec translocase system (Driessen & Nouwen, 2008). All proteins processed by the Sec translocase contain an N-terminal signal peptide sequence which, after translocation, is targeted by one of two signal peptidases (SPases). Type-I SPases recognize an AxAA cleavage site (van Roosmalen *et al.*, 2004), while type-II SPases recognize an L-x-x-C, or lipobox, cleavage site (Sutcliffe & Harrington, 2002). Using SignalP 4.1 (Petersen *et al.*, 2011), 30 of the 37 identified proteins have predicted cleavage sites through either the type-I SPase or the type-II SPase pathway.

**Table 2.** Proteins extracted through modified exposure to LiCl

ORF	Protein description	Predicted molecular mass (kDa)	SPase target	Amino acid coverage	GRAVY score	Predicted SPase cleavage site†	Unique spectral count‡
**Extracellular proteins**							
LBA0695	Putative bacterial Ig-like domain protein	62	SPI	75 % (410/550)	−0.58	VSA-AD (37–38)	141
LBA1029	Putative S-layer protein	43	SPI	78 % (300/385)	−0.41	VQA-AT (37–38)	95
LBA0512	SlpX	54	SPI	65 % (324/499)	−0.58	VQA-DT (30–31)	68
LBA1567	Aminopeptidase	57	SPI	67 % (339/505)	−0.59	AQA-AA (27–28)	66
LBA0222	Putative uncharacterized protein	30	SPI	54 % (152/282)	−0.78	AHA-KG (39–40)	61
LBA0191	Putative fibronectin domain protein	52	SPI	71 % (329/463)	−0.59	VQA-GT (24–25)	60
LBA0864	Putative uncharacterized protein	55	SPI	64 % (316/497)	−0.52	AQA-QH (25–26)	52
LBA1568	Putative surface protein	39	SPI*	66 % (233/353)	−0.38	Ambiguous	46
LBA1539	Putative uncharacterized protein	19	SPI	71 % (122/171)	−0.29	ANA-AS (28–29)	41
LBA1006	Penicillin-binding protein	41	SPI	76 % (276/364)	−0.46	VHA-AY (26–27)	36
LBA0176	*N*-Acetylmuramidase	45	SPI	43 % (176/409)	−0.68	VSA-AT (38–39)	21
LBA0494	Putative surface exclusion protein	40	SPI	47 % (168/355)	−0.53	VQA-AS (32–33)	18
LBA0177	Autolysin, amidase	41	SPI	44 % (160/364)	−0.57	VQA-DS (30–31)	18
LBA0046	Putative uncharacterized protein	13	SPI	53 % (62/118)	−0.29	TQA-AS (30–31)	12
LBA1079	Putative cell surface protein	23	SPI	39 % (79/202)	−0.38	VNA-TT (29–30)	12
**Extracellular proteins (predicted to be sec-attached)**							
LBA1578	Putative serine protease	78	SPI	84 % (583/694)	−0.62	VKA-AD (34–35)	202
LBA0169	SlpA	47	SPI	63 % (278/444)	−0.25	VSA-AT (31–32)	65
LBA0858	Penicillin-binding protein	42	SPI	67 % (248/369)	−0.43	VNA-KV (30–31)	43
LBA1426	Putative uncharacterized protein	28	SPI	63 % (159/252)	−0.38	VQA-AT (34–35)	39
LBA1690	Putative surface exclusion protein	31	SPI	74 % (207/280)	−0.65	NQE-DN (30–31)	35
LBA1207	Putative enterolysin A	24	SPI	65 % (139/213)	−0.42	VSA-DT (30–31)	26
LBA1661	Putative membrane protein	20	SPI	48 % (86/180)	−0.45	VQA-AT (37–38)	25
LBA1227	Putative uncharacterized protein	21	SPI	60 % (109/182)	−0.59	VNA-ST (33–34)	23
LBA1225	Putative bacterial Ig-like domain protein	57	SPI	19 % (95/501)	−0.51	VLA-CS (27–28)	10
**Lipid-anchored proteins**							
LBA0197	OppA – oligopeptide binding protein	65	SPII	63 % (366/585)	−0.58	ALA-AC (21–22)	53
LBA1641	Glycerol-3-phosphate ABC transporter	47	SPII	68 % (293/433)	−0.48	SSS-SS (32–33)	44
LBA1588	PrsA/PrtM – peptidylprolyl isomerase	33	SPII	64 % (193/300)	−0.58	STA-AS (33–34)	38
LBA0014	Putative alkylphosphonate ABC transporter	35	SPII	60 % (187/313)	−0.38	TSA-SS (31–32)	29
LBA0585	Glycerol-3-phosphate ABC transporter	48	SPII	34 % (147/432)	−0.47	NSS-ST (31–32)	16
LBA1497	Putative uncharacterized protein	36	SPII	37 % (125/336)	−0.77	SQG-NS (26–27)	11
**N-terminally anchored proteins (no predicted cleavage site)**							
LBA0223	CdpA – cell separation protein	64	SPI*	62 % (372/599)	−0.53	−	66
LBA0805	Penicillin-binding protein	79	SPI*	53 % (379/720)	−0.37	−	49
LBA1010	Putative secreted protein	45	SPI*	29 % (115/401)	−0.58	−	14
**Intracellular or moonlighting proteins**							
LBA0851	LysA – diaminopimelate decarboxylase	35	−	70 % (226/323)	−0.22	−	32
LBA0040	Putative uncharacterized protein	10	−	74 % (64/87)	−0.98	−	15
LBA0297	RpsC – 30S ribosomal protein	25	−	44 % (99/224)	−0.54	−	14
LBA0698	Glyceraldehyde 3-P dehydrogenase	36	−	30 % (102/338)	−0.09	−	10

* Proteins predicted to have an SPase type-I target with unknown cleavage site.

† The amino acid sequence of predicted SPase cleavage is displayed along with the sequence position in parenthesis.

‡ The number of unique spectra for each protein is used as a semiquantitative measure of protein abundance. The proteins are ordered from highest to lowest unique spectral counts.

With reference to LocateP and the LAB-Secretome database (Zhou *et al.*, 2008, 2010), proteins were sorted by their predicted subcellular locations. Twenty-four of the 37 proteins in the fraction are predicted to be extracellular with predicted type-I SPase-mediated N-terminal cleavage sites (Table 2). Notably, nine of these extracellular proteins are predicted, via hidden Markov models, to remain N-terminally associated with the cell membrane despite predicted cleavage sites. While these so-called ‘sec-attached’ proteins have been proteomically described in *Bacillus subtilis* (Tjalsma & van Dijl, 2005), there has been no work demonstrating the existence of such proteins in *Lactobacillus* species to date. In fact, the exact method by which such proteins avoid processing through SPase activity has not been elucidated (Dreisbach *et al.*, 2011; Tjalsma & van Dijl, 2005). Six of the remaining 13 proteins are predicted to be lipid-anchored proteins, mediated through type-II SPase activity; three are proteins predicted to be N-terminally anchored to the cell membrane due to the lack of predicted cleavage sites; and four are predicted to be intracellular or non-classically secreted proteins (Table 2).

To deduce which of the proteins in the LiCl-extracted fraction may be SLAPs, we compared the protein sequences against the deduced proteomes of closely related S-layer-forming (*L. helveticus*, *L. crispatus* and *L. amylovorus*) and non-S-layer-forming (*L. gasseri*, *L. johnsonii*, and *L. delbrueckii* subsp. *bulgaricus*) lactobacilli using blastp (Table 3). The majority of the extracellular proteins (18/24), including those described as potentially sec-attached, demonstrate high levels of sequence identity to the corresponding proteins in S-layer-forming lactobacilli, but with either weak sequence similarity or no orthologues in the non-S-layer-forming lactobacilli, whereas the lipid-anchored proteins and the intracellular proteins demonstrate equally high sequence identity in both the S-layer-forming and non-S-layer-forming species of *Lactobacillus* examined. There were eight proteins with highly inferred homology in the S-layer-forming species with no hits in all three non-S-layer-forming species. Furthermore, 19 proteins demonstrate a highly inferred homology in the S-layer-forming species with no hit in at least two of the non-S-layer-forming species. We propose that these 17 proteins (excluding SlpA and SlpX) are candidate SLAPs, given their absence in non-S-layer-forming *Lactobacillus* species that are closely related to *L. acidophilus*.

**Table 3.** Homology search of potential SLAPs to proteins in S-layer-forming and non-S-layer-forming lactobacilli For each protein alignment, highest % identity score was presented for the six *Lactobacillus* species listed. blast analyses were set to display alignments with E-values ≤1×10^−6^. —, no hits; no shading, 1-25%; light grey shading, 26-50%; dark grey shading, 51-75%; black shading, 76-100%.

		S-layer-forming lactobacilli			Non-S-layer-forming lactobacilli		
ORF	Protein description	*L. helveticus*	*L. crispatus*	*L. amylovorus*	*L. gasseri*	*L. johnsonii*	*L. delbrueckii*
**Extracellular proteins**							
LBA0695*	Putative bacterial Ig-like domain protein	434/543 (80 %)	429/543 (79 %)	455/543 (84 %)	−	−	167/482 (35 %)
LBA1029*	Putative S-layer protein	145/377 (38 %)	134/371 (36 %)	87/255 (34 %)	−	−	−
LBA0512	SlpX	385/511 (75 %)	313/446 (70 %)	314/517 (54 %)	−	−	52/126 (41 %)
LBA1567*	Aminopeptidase	414/505 (82 %)	405/505 (80 %)	407/505 (81 %)	−	−	−
LBA0222*	Putative uncharacterized protein	2112/129 (86 %)	190/285 (67 %)	110/126 (87 %)	−	−	−
LBA0191*	Putative fibronectin domain protein	379/464 (82 %)	367/464 (79 %)	134/387 (35 %)	−	−	−
LBA0864*	Putative uncharacterized protein	196/474 (41 %)	346/502 (69 %)	401/496 (81 %)	−	−	226/517 (44 %)
LBA1568*	Putative surface protein	252/325 (78 %)	252/325 (78 %)	252/325 (78 %)	−	−	42/133 (32 %)
LBA1539*	Putative uncharacterized protein	111/176 (63 %)	115/173 (66 %)	115/175 (66 %)	−	−	−
LBA1006	Penicillin-binding protein	303/365 (83 %)	304/368 (83 %)	300/364 (82 %)	75/305 (25 %)	65/234 (28 %)	152/336 (45 %)
LBA0176	*N*-Acetylmuramidase	324/409 (79 %)	300/409 (73 %)	334/410 (81 %)	124/273 (45 %)	123/272 (45 %)	190/414 (46 %)
LBA0494	Putative surface exclusion protein	252/356 (71 %)	238/355 (67 %)	241/357 (68 %)	−	26/79 (33 %)	113/257 (44 %)
LBA0177*	Autolysin, amidase	280/364 (77 %)	271/365 (74 %)	284/369 (77 %)	−	−	166/385 (43 %)
LBA0046*	Putative uncharacterized protein	81/101 (80 %)	92/118 (78 %)	90/118 (76 %)	−	−	−
LBA1079	Putative cell surface protein	76/165 (46 %)	78/167 (47 %)	164/202 (81 %)	89/190 (47 %)	−	89/234 (38 %)
**Extracellular proteins (predicted to be sec-attached)**							
LBA1578*	Putative serine protease	135/438 (31 %)	133/481 (28 %)	130/420 (31 %)	−	−	−
LBA0169	SlpA	323/446 (72 %)	250/454 (59 %)	239/488 (49 %)	−	−	53/131 (40 %)
LBA0858	Penicillin-binding protein	265/366 (72 %)	260/369 (70 %)	275/368 (75 %)	77/276 (28 %)	78/313 (25 %)	159/338 (47 %)
LBA1426*	Putative uncharacterized protein	154/263 (59 %)	153/257 (60 %)	148/260 (57 %)	−	−	−
LBA1690	Putative surface exclusion protein	82/90 (91 %)	230/280 (82 %)	232/282 (82 %)	−	82/310 (26 %)	103/283 (36 %)
LBA1207	Putative enterolysin A	117/147 (80 %)	150/211 (71 %)	130/184 (71 %)	63/169 (78 %)	65/170 (38 %)	100/164 (61 %)
LBA1661	Putative membrane protein	68/140 (49 %)	127/183 (69 %)	138/182 (76 %)	76/192 (40 %)	80/184 (43 %)	−
LBA1227*	Putative uncharacterized protein	99/140 (71 %)	137/182 (75 %)	70/96 (73 %)	−	−	48/157 (31 %)
LBA1225*	Putative bacterial Ig-like domain protein	189/294 (64 %)	305/501 (61 %)	195/510 (38 %)	−	−	90/368 (24 %)
**Lipid-anchored proteins **							
LBA0197*	OppA – oligopeptide binding protein	476/541 (88 %)	484/539 (90 %)	490/543 (90 %)	−	−	332/543 (61 %)
LBA1641	Glycerol-3-phosphate ABC transporter	374/433 (86 %)	390/433 (90 %)	403/434 (93 %)	310/433 (72 %)	309/433 (71 %)	357/433 (82 %)
LBA1588	PrsA/PrtM – peptidylprolyl isomerase	276/300 (92 %)	278/296 (94 %)	284/296 (96 %)	214/296 (72 %)	212/296 (72 %)	167/298 (56 %)
LBA0014	Putative alkylphosphonate ABC transporter	278/308 (90 %)	298/311 (96 %)	300/314 (96 %)	218/308 (71 %)	220/308 (71 %)	176/309 (57 %)
LBA0585	Glycerol-3-phosphate ABC transporter	383/433 (94 %)	369/434 (85 %)	404/434 (93 %)	250/436 (57 %)	291/432 (67 %)	258/433 (60 %)
LBA1497*	Putative uncharacterized protein	246/338 (73 %)	250/341 (73 %)	253/337 (75 %)	−	−	90/368 (24 %)
**N-terminally anchored proteins (no predicted cleavage site)**							
LBA0223*	CdpA – cell separation protein	213/308 (69 %)	337/602 (56 %)	392/583 (67 %)	−	−	74/232 (32 %)
LBA0805	Penicillin-binding protein	629/720 (87 %)	609/720 (85 %)	642/720 (89 %)	449/722 (62 %)	445/720 (62 %)	434/722 (60 %)
LBA1010	Putative secreted protein	323/401 (81 %)	328/401 (82 %)	324/401 (81 %)	193/390 (49 %)	185/363 (51 %)	190/405 (47 %)
**Intracellular or moonlighting proteins**							
LBA0851	LysA – diaminopimelate decarboxylase	353/432 (82 %)	336/432 (78 %)	366/432 (85 %)	−	332/431 (77 %)	282/431 (65 %)
LBA0040	Putative uncharacterized protein	74/87 (85 %)	−	82/87 (94 %)	52/85 (61 %)	−	45/86 (52 %)
LBA0297	RpsC – 30S ribosomal protein	220/224 (98 %)	221/224 (99 %)	219/224 (98 %)	192/223 (86 %)	193/223 (87 %)	189/224 (84 %)
LBA0698	Glyceraldehyde 3-P dehydrogenase	296/338 (88 %)	299/338 (88 %)	298/338 (88 %)	314/338 (93 %)	314/338 (93 %)	317/338 (93 %)

* Candidate SLAP.

While both SlpX and SlpA were found to be present in the SLAP fraction, they appeared at a significantly lower concentration compared with the other proteins (Table 2). For reference of comparison, the standard LiCl extraction protocol was performed along with the modification presented in this study (Fig. 2b). It is clear that the method presented in this study recovered more proteins than the standard method. Of the 37 proteins identified, 21 are annotated as putative or uncharacterized proteins of unknown function. These include proteins with putative fibronectin-binding domains, putative surface proteins, bacterial Ig-like domain proteins, putative surface exclusion proteins, uncharacterized ABC transporters, a 78 kDa putative serine protease and a putative S-layer protein (LBA1029).

### Deletion of *lba-1029* from the chromosome of *L. acidophilus* NCFM

**Fig. 3.**
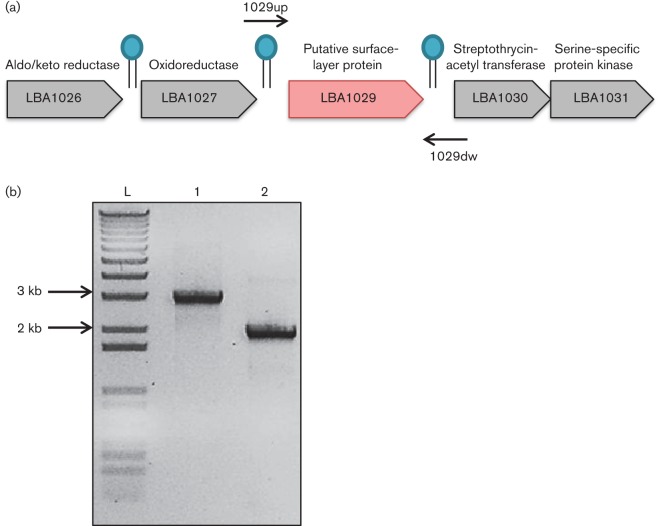
(a) The *lba-1029* ORF in chromosomal context. It is flanked by genes encoding an aldo/keto reductase, an oxidoreductase, streptothrycin-acetyl transferase and a serine-specific protein kinase. Note the predicted hairpin Rho-independent terminators flanking *lba-1029*. The forward and reverse primers used to confirm the deletion are indicated. (b) Gel electrophoresis using the forward and reverse primers noted in (a) on WT reference strain (lane 1) compared with NCK2258 (lane 2). Deletion of 1155 bp from *lba-1029* in the chromosome of *L. acidophilus*, reflected in lane 2, was confirmed by sequencing.

LBA1029 was observed to be a prevalent protein in the LiCl fraction. Examining annotated sequence data from *L. acidophilus* NCFM, we found that *lba-1029* encodes an uncharacterized protein, annotated as a putative S-layer protein. Notably, this protein was selected for functional analysis because of its apparent singularity to *L. acidophilus* compared with closely related S-layer- and non-S-layer-forming lactobacilli (Table 3). In fact, the deduced protein sequence of LBA1029 demonstrated low sequence identity to proteins in the dairy starter culture *L. helveticus* H10 (38 % sequence identity) and the vaginal commensal *L. crispatus* ST1 (36 %), both of which are S-layer-forming members of the Group A acidophilus complex. This 385 aa residue protein of unknown function has a predicted N-terminal signal peptide cleavage site between two alanine residues at positions 37 and 38. Furthermore, *lba-1029* is flanked by two hairpin terminators, suggesting monocistronic mRNA expression and control (Fig. 3a).

To assess the roles these putative SLAPs may play in cell function and immunomodulatory interaction, a Δ*lba-1029* strain was created and phenotypically characterized. The *lba-1029* gene was deleted from the chromosome using a *upp*-based counterselection gene replacement system (Fig. 3b). A colony containing the in-frame deletion of *lba-1029* (Δ*lba-1029*) was confirmed via sequencing and designated NCK2258. When the SLAPs of NCK2258 were profiled (Fig. 2, lane 2) and identified through LC-MS/MS, LBA1029 was not found in the fraction (data not shown), further confirming the absence of LBA1029 from the cell envelope of NCK2258.

### Phenotypic characterization of Δ*lba-1029* mutant as pertaining to probiotic functionality

Comparative analysis between NCK1909 (wild-type reference) and NCK2258 (Δ*lba-1029*) was used to characterize the function of LBA1029. NCK2258 showed no difference in growth in MRS medium or cell morphology under the light microscope compared with NCK1909. Likewise, mutant cells settled to the bottom of tubes in a similar fashion to NCK1909 when grown in planktonic culture, suggesting no difference in the aggregative properties between the two strains.

Assays for simulated gastric and small intestinal juices, adhesion to a Caco-2 epithelial cell line, and bile tolerance were also performed. Survival through the gastrointestinal tract was evaluated *in vitro* through exposure to simulated gastric juice and simulated small intestinal juice over 1.5 and 5 h, respectively. There was no significant difference in the survival rate between NCK2258 and the NCK1909 reference strain (Fig. S1, available in *Microbiology* Online). The Caco-2 epithelial cell line was employed for *in vitro* analysis of bacterial adherence to intestinal epithelia. Compared with the reference strain, NCK2258 showed insignificant changes in the adherence to Caco-2 (Fig. S2). Finally, NCK2258 and the NCK1909 reference strain were exposed to 0.3 and 0.5 % bile (Oxgall) to assay bile tolerance, but no difference was observed between the strains (Fig. S3).

### LBA1029 contributes to pro-inflammatory response through the induction of TNF-α

Previous work on the S-layer of *L. acidophilus* NCFM demonstrated a role of SlpA in modulating the host immune system (Konstantinov *et al.*, 2008). To test the immunomodulatory action of the LBA1029 protein, a bacterial/murine DC co-incubation assay was performed. After co-incubation for 24 h, the cytokines TNF-α, IL-6, IL-10 and IL-12 were quantified using ELISA. Both TNF-α and IL-6 were measured as an indicator of a general pro-inflammatory response. IL-10, however, was measured as a marker of an anti-inflammatory response via the downregulation of a Th1 cell response. Conversely, IL-12 was measured as a pro-inflammatory response via the activation of Th1 cells. Three independent assays were performed in duplicate for each cytokine.

**Fig. 4.**
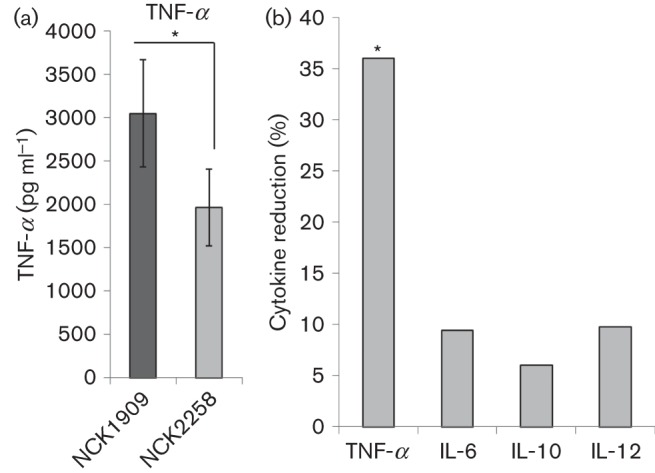
(a) Induction of TNF-α in murine DCs after co-incubation with NCK1909 or NCK2258 as measured by ELISA. Three independent biological replicates were performed with each strain in duplicate, aiming for an approximate bacterial to DC ratio of 10 : 1. Using univariate ANOVA, the reduction in TNF-α induction between NCK1909 and NCK2258 was significant (*P* = 0.006). Error bars show sd among the replicates. (b) Per cent cytokine reduction of NCK2258 compared with NCK1909 for the cytokines TNF-α, IL-6, IL-10 and IL-12. The induction of IL-6, IL-10 and IL-12 was not significantly different between NCK2258 and NCK1909.

For each co-incubation, bacterial cells were diluted and exposed to DCs at a ratio of approximately 10 : 1. Notably, there was a significant decrease (*P* = 0.006) in TNF-α production for DCs co-incubated with NCK2258 compared with the parental reference strain, NCK1909 (Fig. 4a). There was no significant difference between DCs co-incubated with NCK2258 and the NCK1909 reference with regard to IL-6, IL-10 and IL-12 production (Fig. 4b). Ultimately, NCK2258 demonstrated a 36 % reduction in TNF-α induction (Fig. 4b), suggesting that LBA1029 contributes to a pro-inflammatory response via the induction of TNF-α.

## Discussion

This study utilized a method modified from a standard LiCl S-layer extraction to isolate proteins associated with the primary S-layer fraction and identify extracellular proteins and putative SLAPs in *L. acidophilus* NCFM. Thity-seven proteins were identified and reported. One such protein, LBA1029, was eliminated via deletion of the gene from the chromosome. While the absence of LBA1029 did not seem to affect survival in simulated gastric juice, simulated small intestinal juice, bile tolerance or affinity to Caco-2 epithelial cells, it did demonstrate important capacity for immunomodulation through murine DCs. The 36 % reduction of TNF-α production by DCs co-incubated with the LBA1029 deficient strain compared with its parent strain suggests that the protein is pro-inflammatory through the TNF-α pathway.

Of the 37 reported proteins, 30 have predicted cleavage sites for secretion, of which 24 are predicted to be extracellular and six are lipid-anchored. Three of the remaining seven have an N-terminal transmembrane hydrophobic anchor (with no cleavage site) and the final four are intracellular, potentially non-classically secreted moonlighting proteins (Table 2). Both LocateP (Zhou *et al.* 2008) and the LAB-Secretome database (Zhou *et al.*, 2010) reported nine of the 24 extracellular proteins as ‘sec-attached’. These sec-attached proteins, discovered in *Bacillus subtilis* (Tjalsma & van Dijl, 2005), are proteins that have predicted cleavage sites but avoid Sec-pathway SPase cleavage to remain N-terminally anchored to the cell membrane. The method by which these proteins avoid SPase activity for Sec processing has yet to be elucidated (Dreisbach *et al.*, 2011; Tjalsma & van Dijl, 2005). Notably, sec-attached proteins have not been proteomically characterized in any *Lactobacillus* species to date. The basis by which the LAB proteomes were analysed for the presence of sec-attached proteins used hidden Markov modelling comparing 63 cleaved proteins (extracellular) with 53 un-cleaved proteins (sec-attached) from *B. subtilis* and other *Bacillus* orthologues (Zhou *et al.*, 2008). While this hidden Markov modelling scoring model is useful for processing large proteomic datasets, such as those from the LAB-Secretome database, it is important to acknowledge the possibility of incorrect predictions. For example, SlpA was predicted to be sec-attached despite the fact that it has been visually and biochemically characterized as the self-assembling constituent of the *L. acidophilus* S-layer (Boot *et al.*, 1995, 1996; Boot & Pouwels, 1996). Due to this incongruity and because these nine predicted sec-attached proteins were isolated using LiCl, we consider these proteins to be extracellular.

Interestingly, four of the 37 proteins identified (Table 2) in the LiCl precipitate do not have predicted secretion pathways: a diaminopimelate decarboxylase, 30S ribosomal protein S3, an uncharacterized protein and a glyceraldehyde 3-phosphate dehydrogenase (GAPDH). Because the cells were dialysed at stationary phase, it is possible that limited cell lysis may have begun by the time cells were treated with LiCl, allowing small amounts of intracellular and membrane-anchored proteins to be recovered non-specifically along with the S-layer. However, it is also possible that these intracellular proteins are non-classical, extracellular ‘moonlighting’ proteins (Jeffery, 1999). GAPDH, for example, is a moonlighting protein which mediates microbe–host interactions in lactic acid bacteria (Katakura *et al.*, 2010; Kinoshita *et al.*, 2008; Sánchez *et al.*, 2009). In fact, the GAPDH of *L. plantarum* LA 318 has demonstrated adherence capabilities to human colonic mucin (Kinoshita *et al.*, 2008).

Of particular interest in this study is the isolation and identification of possible SLAPs in the cell-surface proteome of *L. acidophilus* NCFM. Note that the *in vivo* localization or association of these SLAPs in relation to the S-layer has not been fully demonstrated, and is based on co-extraction following LiCl treatment. To select candidate SLAPs from the fraction of secreted proteins identified through LiCl extraction, we aligned the deduced protein sequences to the deduced proteomes of closely related S-layer- and non-S-layer-forming *Lactobacillus* species (Table 3). The analysis revealed that the 24 extracellular proteins had high sequence identity to the corresponding orthologues in S-layer-forming lactobacilli and either no or low sequence identity to the non-S-layer-forming lactobacilli. In contrast, the lipid-anchored proteins, many of which are ABC sugar transporters, and the intracellular proteins shared inferred homology in both S-layer- and non-S-layer-forming *Lactobacillus* species. Based on this *in silico* analysis, the proteins that are most likely SLAPs are the 17 extracellular proteins with no hits in two or three of the non-S-layer-forming lactobacilli. Further work will be required to characterize the specific localization of these SLAPs and the Slp subunits with which they interact. Furthermore, there should be a distinction between the organizational definitions of SLAPs in the context of this study and the SLAP Pfam domain (PF03217) designated for bacterial S-layer proteins (Boot *et al.*, 1995).

To our knowledge, SLAPs have not been identified in any organism using the method of this study. However, in the S-layer-forming pathogen *Bacillus anthracis*, 22 *B. anthracis* S-layer proteins (BSLs) have been identified and characterized (Kern & Schneewind, 2008, 2010; Kern *et al.*, 2012; Lunderberg *et al.*, 2013). While these proteins are described as S-layer associated in recent publications (Kern *et al.*, 2012; Lunderberg *et al.*, 2013), these proteins were localized *in silico* based on N-terminal SLH domains and were originally designated Slps. In contrast, the SLAPs observed in the present study were found to be constituents of the S-layer after translational expression and secretion. An important distinction should therefore be made between the methodology of this study and the methodology for identifying BSLs of *B. anthracis.* Application of this study’s methodology to *B. anthracis* may yield novel SLAPs that are not tethered to the S-layer through SLH domains.

The SLAPs in *L. acidophilus* NCFM offer potential in understanding cell envelope biology and function, as well as illuminating important factors pertaining to probiotic function. A cell division protein CdpA, which has previously been functionally characterized (Altermann *et al.*, 2004), was found to be a prevalent protein in the SLAP fraction. CdpA, which may be a SLAP due to its low sequence identity in the non-S-layer-forming lactobacilli (Table 3), has important roles in cell-wall processing during growth and cell–cell separation. This finding substantiates the prediction that certain SLAPs, especially the putative cell division proteins, aminopeptidases and penicillin-binding proteins, may play a role in cell growth, cell turnover and cell envelope function. A great deal of work has already been done pertaining to probiotic functions in *L. acidophilus* NCFM. This includes work on adherence factors (Buck *et al.*, 2005), probiotic sugar utilization (Barrangou *et al.*, 2003) and bacteriocin production (Dobson *et al.*, 2007). However, there are still many factors of the probiotic mechanism that have not been fully elucidated. Because of their localization to the cell surface, the SLAPs identified in this study are candidate mediators of probiotic function. Furthermore, access to microarray data pertaining to acid tolerance (Azcarate-Peril *et al.*, 2004), bile tolerance (Pfeiler *et al.*, 2007) and oligosaccharide utilization (Barrangou *et al.*, 2003), could offer insight into the role that these extracellular proteins may play in probiotic survival, persistence and immunomodulation in the host gastrointestinal tract.

Understanding the role of intestinal microbiota in gut homeostasis has been regarded with great interest given the prevalence of inflammatory bowel disease, such as ulcerative colitis, and Crohn’s disease (MacDonald & Monteleone, 2005). Recent work regarding cell surface components of *L. acidophilus* NCFM, such as lipoteichoic acid and SlpA, has demonstrated regulation of colonic inflammation and T-cell functionality, respectively (Konstantinov *et al.*, 2008; Mohamadzadeh *et al.*, 2011). Given their localization to the outermost layer of the cell envelope, SLAPs are ideal candidates for studying the immunomodulatory interaction between *L. acidophilus* and the gut immune system. The findings of this study support this observation, given that LBA1029 exhibited an effect on immunomodulatory properties through the induction of TNF-α. Exploring the SLAPs of *L. acidophilus*, as well as those of other S-layer-forming commensal bacteria, will be important in understanding the full context of the interaction between gut epithelial cells, gut immune system and intestinal microbiota.

Beyond *L. acidophilus*, this study highlights further potential in understanding S-layer and cell envelope function of other S-layer-forming bacteria and archaea. In certain S-layer-forming pathogens, such as *B. anthracis* (Kern & Schneewind, 2010), *Rickettsia* species (Walker *et al*., 2003), *Aeromonas* *salmonicida* (Kay *et al.*, 1984), *Campylobacter fetus* (Blaser *et al.*, 1988) and *Clostridium difficile* (Calabi *et al.*, 2002), S-layer and cell surface components are important pathogenicity factors. Studying potential SLAPs with the cell surface proteome in these and other S-layer-forming pathogens may offer further insight into pathogenicity. The hyperthermophilic archaea that form S-layers, such as *Methanococcus* and *Methanothermus* species, are of certain biotechnological interest. In particular, the hexagonal S-layers of these archaea have been regarded for their potential uses in nanotechnology because of their heat stability and the ability to self-assemble (Sleytr & Sára, 1997). Examining potential SLAPs in these and other extremophilic archaea could explicate cell envelope function, as well as potentiate the discovery of important thermostable proteins for use in biotechnological applications.

Overall, the characterization of SLAPs significantly expands the opportunities to understand the probiotic activities and immunomodulatory actions of *L. acidophilus* NCFM. Likewise, SLAPs may be of particular interest in other members of the genus *Lactobacillus*. Comparing the SLAPs of *L. acidophilus* with the proteomes of other S-layer-forming lactobacilli, such as the dairy starter *L. helveticus* and the vaginal commensal *L. crispatus*, could provide key ecological and evolutionary insights. Novel proteinases or proteases could be identified as SLAPs in the cell surface proteome of *L. helveticus* that may be important in the cheese ripening process. Moreover, SLAPs could be important factors of adherence and retention for *L. crispatus* in the vaginal mucosa. Ultimately, the proteins identified in this study are potentially novel extracellular proteins, some of which may be associated with the bacterial S-layer. These proteins afford the possibility to functionally characterize bacterial S-layers and will provide important insights into the architecture and physiology of bacterial cell surfaces.
